# Post-discharge occurrence of surgical site infections after hip or knee arthroplasty surgery in Poland, a population-based study

**DOI:** 10.1038/s41598-023-43111-z

**Published:** 2023-09-24

**Authors:** Mateusz Gajda, Paulina Gajda, Agnieszka Pac, Barbara Gryglewska, Marcin Wojnarski, Anna Różańska, Inga Lipińska-Tobiasz, Jadwiga Wójkowska-Mach

**Affiliations:** 1 Department of Microbiology, Faculty of Medicine, Jagiellonian University Medical College, Czysta 18 St., 31-121 Kraków, Poland; 2 Doctoral School of Medical and Health Sciences, Jagiellonian University Medical College, Łazarza 16 St., 31-530 Kraków, Poland; 3 Chair of Epidemiology and Preventive Medicine, Department of Epidemiology, Jagiellonian University Medical College, Kopernika 7 St., 31-034 Kraków, Poland; 4 Department of Internal Medicine and Gerontology, Jagiellonian University Medical College, Jakubowskiego 2 St., 30-886 Kraków, Poland; 5 Faculty of Medicine, Jagiellonian University Medical College, St. Anna 12 St., 33-332 Kraków, Poland; 6 Department of Orthopedics and Traumatology, Hospital in Proszowice, Kopernika 13 St, 32-100 Proszowice, Poland

**Keywords:** Microbiology, Health care, Risk factors

## Abstract

Arthroplasty is a common procedure improving functioning of patients and their quality of life. Infection is a serious complication that determines subsequent management of the prosthesis and the patient. The aim of the study was to investigate the incidence of post-discharge surgical site infections (SSI) and their risk factors. A retrospective analysis of an anonymized database from the National Health Found for 2017 of 56,068 adult patients undergoing hip replacement surgery (HPRO) and 27,457 patients undergoing knee replacement surgery (KPRO). The cumulative incidence of post-discharge SSI was 0.92% for HPRO and 0.95% for KPRO. The main risk factors for hip SSI were male gender, diseases of hematopoietic, musculoskeletal and nervous system. The risk factor for knee SSI was male gender. All comorbidities significantly increased the risk of SSI. The ICU stay and antibiotics administered at discharge in studied population increased the risk of detection of SSI after HPRO and KPRO by up to four and seven times, respectively. For both procedures rehabilitation after surgery and total endoprosthesis decreased incidence of SSIs. The lower experience of the center was related to higher SSI incidence in HPRO in primary (1.5% vs. 0.9%) and in revision surgeries (3.8% vs. 2.1%), but in KPRO, lower experience only in primary surgeries was significantly associated with SSI. The cumulative incidence of post-discharge SSI in Poland is higher than in other European countries. Special attention should be paid to patients with chronic diseases.

## Introduction

Arthroplasty procedures are performed more and more frequently in the world due to age-related degeneration of joints and the general tendency to shift the age of the population and due to the impact of arthroplasty on quality of life, they are the dominant method of treating this type of disease^1^. In Poland, more than 83,000 hip or knee arthroplasty were performed in 2017, although among other European countries the number of procedures was much higher^2^. Every surgical procedure, and also arthroplasties, is associated with the risk of complications in the form of surgical site infection (SSI), including deep infection, which requires revision treatment.

Currently, the revision procedure in the case of infection may consist in cleaning and leaving the prosthesis, one-stage replacement of the prosthesis, two-stage replacement of the prosthesis or definitive removal of the prosthes^3^.

 European Center of Disease Prevention and Control (ECDC) data collected in HAI-Net pan-European surveillance system, indicate that SSI incidence rates are high enough in orthopedic and other selected surgical procedures to make them the second most common and most important type of healthcare-associated infection (HAI) in Europe, just like in the USA^4,5^. According to ECDC data for 2017 in European countries (excluding Poland, due to lack of Polish national SSI surveillance system), the incidence rate of SSI in KPRO was 0.5% and in HPRO was 1.0% ). The national discrepancy between hip arthroplasty is wide, from 0.4% for Lithuania and Northern Great Britain to 2.2% for Norway. Analogical data for KPRO showed a discrepancy in the incidence rate from 0.2% for Northern Great Britain to 2.7% for Hungary^6^.

The problem of SSI is important for both, the patient and the health care system. It is associated with reduced quality of life, increased risk of death, prolonged hospital stays and often requiring re-surgical intervention, further increase in mortality and hospitalization costs^7^. According to Natsuhara et al. the immediate complication of all SSI after total hip arthroplasty was associated with a mean all-cause mortality of 5.44% and annual mortality of 4.22%^8^.

So far, in Poland, knowledge about SSI after arthroplasty have been based only on one- or few-centers studies, but the results were very disturbing, e.g., high incidence rates were demonstrated, a two-fold higher after HPRO and four-fold higher after KPRO, additionally the share of the deep SSIs was twice higher than expected^9^. Therefore, the aim of this study was to analyze the post discharge occurrence of deep SSI after hip or knee joint arthroplasty, considering the factors that predispose and protect against its incidence. Cases diagnosed as outpatients or in emergency departments were analyzed. This is the first study of this type conducted in the entire adult population of Polish patients.

## Materials and methods

**Figure 1:**
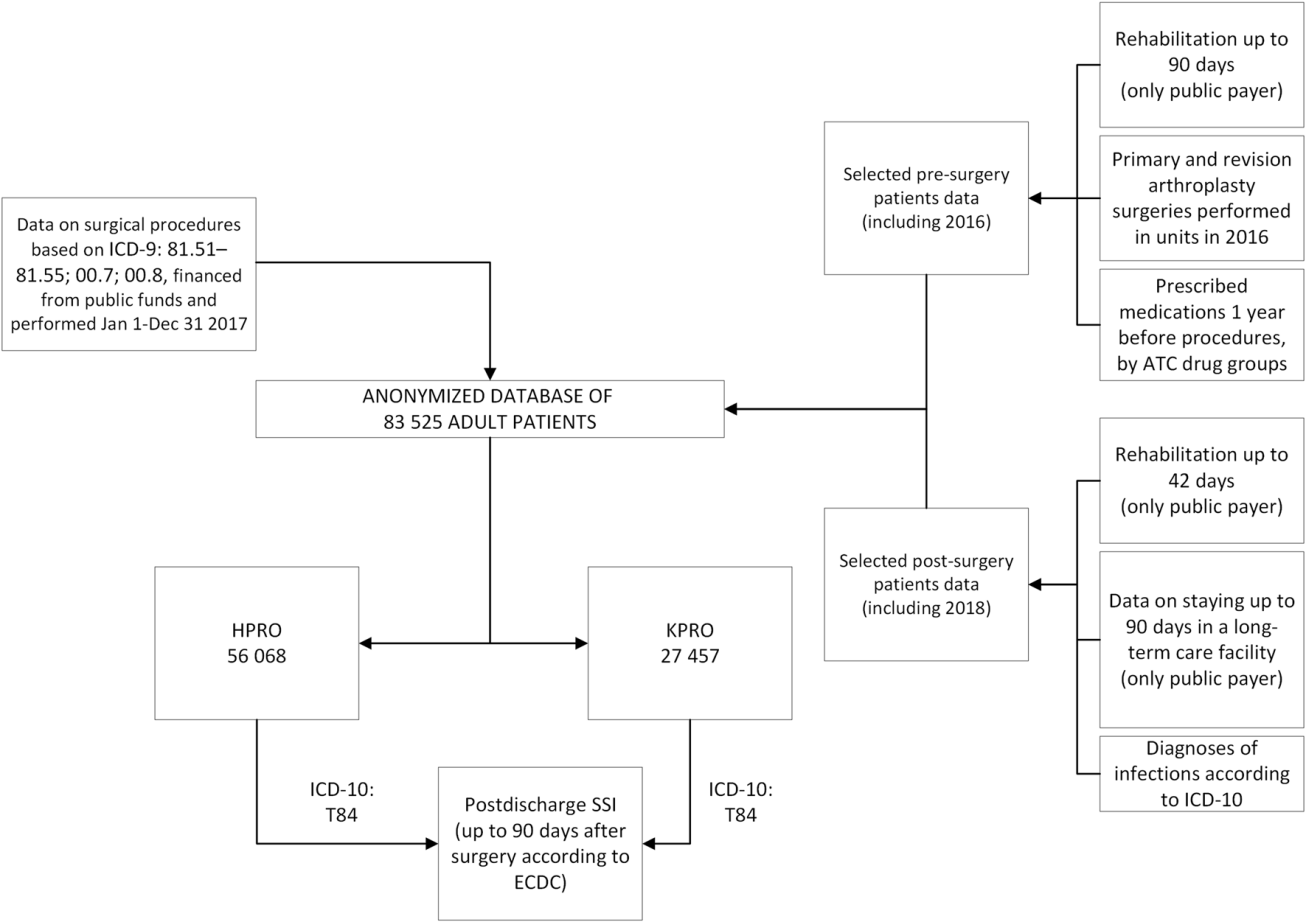
Data included in the database, including time and financial criteria. The structure of the database obtained in cooperation with the Department of Databases and Analytical Tools of the Department of Analyzes and Innovation of the Headquarters of the National Health Fund. *ATC* Anatomical Therapeutic Chemical Classification, *HPRO* hip arthroplasty, *ICD-9* International Classification of Diseases 9th Revision, *ICD-10* International Classification of Diseases 10th Revision, *KPRO* knee arthroplasty, *SSI* surgical site infection.

The analysis was carried out based on an anonymized database, which included 56,068 patients after hip arthroplasty and 27,457 patients after knee arthroplasty, over 18 years of age. The data include information reported by various reporting systems to Narodowy Fundusz Zdrowia (the National Health Fund in Poland) as described on Fig. 1.

The analyzed data included demographic information (sex, age, place of residence), selected elements of preparation for surgical procedures, hospitalization (ICU stay, center experience) and the selected surgery information (divided into hip and knee joints, Supplementary Appendix).

Several assumptions were made to facilitate grouping and statistical analysis of the existing data:

- The cumulative incidence of surgical site infection was defined as the number of new infections that occur in a defined population during a given period
- Patients' burden of particular diseases in the preoperative period was assessed on the basis of information on the use of groups of drugs (prescription only) according to the Anatomical Therapeutic Chemical Classification (ATC) codes
- The general condition of ICU hospitalized patients was assessed with Therapeutic Intervention Scoring System (TISS-76 score), consisting of a daily collection of 76 items (interventions and treatments) scored as Class IV: >  = 40 points, Class III: 20—39 points, Class II: 10 -19 points or Class I: < 10 points
- The experience of the center was assessed based on the number of surgical procedures performed in 2016: units performed less than 100 primary surgeries and\or less than 30 revision surgeries per year were treated as less experienced.
- Post discharge antibiotic therapy was estimated based on information about purchased prescriptions.

The methods and studied population have previously been described in detail^2^.

The authors are unable to assess the knowledge of the staff of the surveyed Polish hospitals on the criteria for diagnosing SSI according to the ECDC definition (Surveillance of surgical site infections and prevention indicators in European hospitals—HAI-Net SSI protocol, version 2.2 from May 2017), and the resulting use of the ICD-10 T84.5 code for identification of SSIs. For the purposes of this study, all SSIs identified in this way were classified as deep or organ/space.

### Statistical analysis

In the statistical analysis, relative and absolute frequencies were used for nominal variables and mean value with standard deviation for quantitative variables (age). For non-normal distribution of data median with interquartile range (IQR) were used. Chi^2^ test and Student's t-test were used to compare the groups of patients (hip vs. knee arthroplasty). The cumulative incidence of SSI after HPRO or KPRO was calculated with respect to the characteristics of the patient as well as of the procedure. The risk of SSIs was assessed in a multivariable logistic regression model with odds ratio (OR) assessment and its 95% confidential intervals (95% CI). In the final model, mostly factors significant in the univariable analysis were included. The analysis was carried out in the International Business Machines Corporation Statistical Package for the Social Sciences, version 26 (IBM Corporation, Armonk, New York, United States). In all analyzes, the significance level was α = 0.05.

### Ethics

All methods were performed in accordance with the relevant guidelines and regulations accepted by the Bioethics Committee of the Jagiellonian University (No. 1072.6120.149.2020 of June 25, 2020).

### Consent to participate

The requirement for informed consent from the study subjects was waived by the Bioethics Committee of the Jagiellonian University (No. 1072.6120.149.2020 of June 25, 2020) due to the retrospective study design and full data anonymization.

## Results

Cumulative incidence of post-discharge SSI was 0.92% (n = 515) for HPRO and 0.95% (n = 262) for KPRO. The ambulatory diagnosis of SSI was made in 48.9% (n = 252) of HPRO patients and in 47.7% (n = 125) after KPRO. In more than 62% cases ambulatory was related to department performing primary surgery. Revision surgery was needed in 72.7% of HPRO cases and 57.5% of KPRO cases. Rehospitalization was in the same unit for both procedures in 93% cases, the median time to readmission was 33 days (IQR 19.5–56.5) for HPRO and 42 days (IQR 28.0–62.0) for KPRO. In both groups, the cumulative incidence of SSI was higher in the urgent admission, as well as in primary surgery and while the reason for the surgery was complications (Table 1). The revision surgery after both procedures was associated with a higher incidence of SSIs after the next surgery.

**Table 1:** Characteristic of hospitalization divided into the HPRO and KPRO group.

Characteristics of the study group		Total (n = 56,068)	SSI percentage, n (%)	p-value	Total (n = 27 457)	SSI percentage, n (%)	p-value
		HPRO			KPRO		
Admission type	Urgent	15,228 (27.2%)	193 (1.3%)	< 0.001	1921 (7.0%)	35 (1.8%)	< 0.001
	Planned	40,690 (72.8%)	319 (0.8%)		25,532 (93.0%)	226 (0.9%)	
Reason for the surgery	Other	456 (0.8%)	7 (1.5%)	< 0.001	139 (0.5%)	1 (0.7%)	< 0.001
	Complication	3582 (6.4%)	207 (5.8%)		1154 (4.2%)	100 (8.7%)	
	Trauma	11,964 (21.3%)	108 (0.9%)		3 (0.0%)	0 (0.0%)	
	Degeneration	40,066 (71.5%)	193 (0.5%)		26,161 (95.3%)	161 (0.6%)	
ICU during first hospitalization	No	55,685 (99.3%)	504 (0.9%)	0.001	27,371 (99.7%)	258 (0.9%)	0.01
	Yes	383 (0.7%)	11 (2.9%)		86 (0.3%)	4 (4.7%)	
Death	No	55,585 (99.1%)	512 (0.9%)	0.81	27,443 (99.9%)	261 (1.0%)	0.13
	Yes	483 (0.9%)	3 (0.6%)		14 (0.1%)	1 (7.1%)	
Teaching unit	No	48,287 (86.1%)	411 (0.9%)	< 0.001	23,940 (87.2%)	216 (0.9%)	0.03
	Yes	7781 (13.9%)	104 (1.3%)		3517 (12.8%)	46 (1.3%)	
Number of primary surgeries	0–100	8043 (14.3%)	124 (1.5%)	< 0.001	14,486 (52.8%)	147 (1.0%)	0.01
	100–200	24,040 (42.9%)	176 (0.7%)		8329 (30.3%)	58 (0.7%)	
	> 200	23,985 (42.8%)	215 (0.9%)		4642 (16.9%)	57 (1.2%)	
Number of revision surgeries	0–30	3877 (45.6%)	146 (3.8%)	< 0.001	1637 (78.9%)	87 (5.3%)	0.62
	> 30	4626 (54.4%)	95 (2.1%)		437 (21.1%)	20 (4.6%)	
Prosthesis type	Primary	52,207 (93.1%)	295 (0.6%)	< 0.001	26,181 (95.4%)	149 (0.6%)	< 0.001
	Revision	3861 (6.9%)	220 (5.7%)		1276 (4.6%)	113 (8.9%)	
Prosthesis range	Total	45,821 (81.7%)	314 (0.7%)	< 0.001	25,494 (92.9%)	202 (0.8%)	< 0.001
	Partial	10,241 (18.3%)	197 (1.9%)		1961 (7.1%)	59 (3.0%)	
Fixation method	Cement	44,115 (78.7%)	380 (0.9%)	0.06	2718 (9.9%)	39 (1.4%)	0.01
	Cementless	9721 (17.3%)	106 (1.1%)		23,734 (86.4%)	209 (0.9%)	
	Hybrid	2226 (4.0%)	25 (1.1%)		1003 (3.7%)	13 (1.3%)	

*SSI* surgical site infection, *HPRO* hip arthroplasty, *KPRO* knee arthroplasty, *ICU* intensive care unit.

General condition of 469 patients who required hospitalization in the ICU was assessed with the TISS score, median TISS score was 40 for both procedures with IQR 36.0; 41.0 for HPRO and 40.0; 40.0 for KPRO.

According to multivariable analysis the odds of SSI for patients needed hospitalization in the ICU were 3 times higher for HPRO and even 5 times higher for KPRO (Table 1).

### Incidence of SSI after HPRO

The median age of the patients was 70.9 years for the females and 65.1 years old for males with higher odds of SSI occurrence (OR = 1.3; 95% CI 1.12–1.63) for the male sex. Higher incidence of SSI was observed in patients with gastrointestinal disease or diabetes, circulatory system diseases, musculoskeletal system diseases, and nervous system diseases during the perioperative period (Table 2). The implications of these chronic conditions were confirmed in the multivariable logistic regression analysis, where all factors excluding gastrointestinal/diabetes diseases significantly increased the incidence of SSI (Table 3).

**Table 2:** Characteristics of the analyzed population, including the percentage of SSI divided into the HPRO and KPRO groups.

Characteristics of the study group		Total (n = 56,068)	SSI percentage, n (%)	p-value	Total (n = 27,457)	SSI percentage, n (%)	p-value
		HPRO			KPRO		
Sex	Female	32,587 (58.1%)	271 (0.8%)	0.01	20,037 (73.0%)	163 (0.8%)	< 0.001
	Male	23,481 (41.9%)	244 (1.0%)		7420 (27.0%)	99 (1.3%)	
Age (years)	< 65	19,822 (35.4%)	173 (0.9%)	0.43	8033 (29.3%)	80 (1.0%)	0.70
	≥ 65	36,246 (64.6%)	342 (0.9%)		19,424 (70.7%)	182 (0.9%)	
Digestive tract diseases and diabetes	No	27,331 (48.7%)	211 (0.8%)	< 0.001	11,290 (41.1%)	76 (0.7%)	< 0.001
	Yes	28,737 (51.3%)	304 (1.1%)		16,167 (58.9%)	186 (1.2%)	
Circulatory system diseases	No	42,317 (75.5%)	279 (0.7%)	< 0.001	19,317 (70.4%)	116 (0.6%)	< 0.001
	Yes	13,751 (24.5%)	236 (1.7%)		8140 (29.6%)	146 (1.8%)	
Musculo-skeletal system diseases	No	22,957 (40.9%)	183 (0.8%)	0.01	9287 (33.8%)	64 (0.7%)	0.002
	Yes	33,111 (59.1%)	332 (1.0%)		18,170 (66.2%)	198 (1.1%)	
Nervous system diseases	No	29,598 (52.8%)	213 (0.7%)	< 0.001	15,109 (55.0%)	121 (0.8%)	0.01
	Yes	26,470 (47.2%)	302 (1.1%)		12,348 (45.0%)	141 (1.1%)	
Rehabilitation prior to surgery	No	53,249 (95.0%)	484 (0.9%)	0.35	25,797 (94.0%)	245 (0.9%)	0.86
	Yes	2819 (5.0%)	31 (1.1%)		1660 (6.0%)	17 (1.0%)	
Antibiotics at discharge	No	53,703 (95.8%)	438 (0.8%)	< 0.001	26,291 (95.8%)	202 (0.8%)	< 0.001
	Yes	2365 (4.2%)	77 (3.3%)		1166 (4.2%)	60 (5.1%)	
Rehabilitation postdischarge	Ambulatory	1758 (3.1%)	7 (0.4%)	< 0.001	1683 (6.1%)	3 (0.2%)	< 0.001
	Stationary	5780 (10.3%)	23 (0.4%)		4461 (16.2%)	27 (0.6%)	
	No	48,530 (86.6%)	485 (1.0%)		21,313 (77.6%)	232 (1.1%)	

*SSI* surgical site infection, *HPRO* hip arthroplasty, *KPRO* knee arthroplasty.

**Table 3:** Multivariable analysis for the SSI endpoint—hip endoprosthesis.

Variable	OR	95% CI		P-value
Age—65+	0.844	0.685	1.041	0.11
Gender—male	1.349	1.120	1.625	0.002
Rehabilitation prior to surgery	1.165	0.803	1.688	0.42
Digestive tract diseases and diabetes	1.147	0.946	1.391	0.16
Hematopoietic system diseases	2.166	1.800	2.605	< 0.001
Circulatory system diseases	1.040	0.843	1.281	0.72
Hormonal diseases	0.938	0.744	1.183	0.59
Cancer	0.765	0.495	1.182	0.23
Musculo-skeletal system diseases	1.219	1.002	1.484	0.048
Nervous system diseases	1.337	1.108	1.614	0.002
Respiratory system diseases	0.954	0.747	1.219	0.71
Intensive care unit	2.034	1.066	3.879	0.03
Urgent admission	0.955	0.753	1.213	0.71
Rehabilitation after surgery				
No	1.00 (ref.)	–	–	–
Ambulatory	0.419	0.198	0.887	0.02
Stationary	0.450	0.295	0.686	< 0.001
Endoprosthesis type				
Cementless	1.00(ref.)	–	–	–
Cement	0.775	0.604	0.995	0.046
Hybrid	1.108	0.731	1.680	0.63
Total endoprosthesis	0.310	0.244	0.394	< 0.001
Discharge antibiotics	3.483	2.712	4.474	< 0.001
LTCF	0.641	0.282	1.453	0.29

*SSI* surgical site infection, *OR* odds ratio, *CI *confidential interval, *LTCF* long term care facility.

The cement endoprosthesis (OR 0.8; 95% CI 0.60–0.99), total endoprosthesis (OR 0.3; 95% CI 0.24–0.39) and rehabilitation after surgery, both ambulatory (OR 0.4; 95% CI 0.19–0.889 and stationary (OR 0.5; 95% CI 0.29–0.69) significantly reduced the incidence of SSI, while stay in the ICU (OR 2.0; 95% CI 1.07–3.88) and discharged with antibiotic therapy (OR 3.5; 95% CI 2.71–4.47) increased.

### Incidence of SSI after KPRO

The median age of the males was 66.6 years old and 69.2 years old for females. The gender of the male significantly increased the incidence of SSI (OR 1.6; 95% CI 1.24–2.10), as well as all studied factors of patients (Table 2). Total endoprosthesis decrease the incidence of SSI (OR 0.3, 95% CI 0.25; 0.48), as well as ambulatory rehabilitation after surgery (OR 0.2, 95% CI 0.06; 0.56, Table 4), but stay in the ICU (OR 3.7; 95% CI: 1.24–10.89) and discharged with antibiotic therapy (OR 5.3, 95% CI 3.91; 7.26) increased the incidence of SSI.

**Table 4:** Multivariable analysis for SSI endpoint—knee endoprosthesis.

	OR	95% CI		P-value
Age—65+	0.949	0.716	1.258	0.72
Gender—male	1.617	1.242	2.104	< 0.001
Rehabilitation prior to surgery	0.910	0.549	1.507	0.71
Digestive tract diseases and diabetes	1.432	1.078	1.901	0.01
Hematopoietic system diseases	2.296	1.768	2.981	< 0.001
Circulatory system diseases	1.161	0.833	1.617	0.38
Hormonal diseases	1.098	0.836	1.443	0.50
Cancer	1.425	0.932	2.179	0.10
Musculo-skeletal system diseases	1.335	0.995	1.790	0.05
Nervous system diseases	1.111	0.860	1.436	0.42
Respiratory system diseases	1.270	0.945	1.706	0.11
Intensive Care Unit	3.668	1.235	10.892	0.02
Urgent admission	1.256	0.846	1.864	0.26
Rehabilitation after surgery				
No	1.00 (ref.)	–	–	–
Ambulatory	0.177	0.056	0.555	0.003
Stationary	0.664	0.440	1.001	0.05
Endoprosthesis type				
Cementless	1.00 (ref.)	–	–	–
Cement	0.948	0.647	1.388	0.78
Hybrid	1.458	0.754	2.820	0.26
Total endoprosthesis	0.345	0.247	0.483	< 0.001
Discharge antibiotics	5.326	3.908	7.261	< 0.001
LTCF	2.310	0.304	17.577	0.42

*SSI* surgical site infection, *OR* odds ratio, *CI* confidential interval, *LTCF* long term care facility.

### Hospital and surgery characteristics and impact on SSI after HPRO or KRRO

There was no difference in SSI incidence rates between teaching and non-teaching hospitals in both surgeries. The lower experience of the center was related to higher SSI incidence in HPRO in primary (1.5% vs. 0.9%) and in revision surgeries (3.8% vs. 2.1%), but in KPRO, lower experience only in primary surgeries was significantly associated with SSI (1.2% vs. 1.0%, respectively, Table 1).

The partial prosthesis increased three times of incidence of SSI in both groups, however, in data there was no information about the result of injury (like neck or femur fractures). The fixation method significantly increased incidence of SSI only for KPRO using the cement and the hybrid method (1.4% and 1.3%, respectively, Table 1).

## Discussion

In our study, we obtained post-discharge SSI cumulative incidence for HPRO—mainly deep or organ/space—at the level of 0.92%, which was comparable to the ECDC data, while in the scope of KPRO we obtained a result of 0.95%, which is almost twice the reported data^6^. But it should be noted that our study only includes post-discharge SSI, while ECDC data include all SSI. So, we can assume that the actual cumulative incidence in Poland is therefore approximately 30% higher than that obtained in this study, according to the data that include the division of SSI into hospital and post-discharge SSI^10^. Therefore, we conclude that the actual percentage of SSI in the population studied, according to other publications, may be approximately 1.5% for HPRO and KPRO^11^.

In Poland, according to data from the Supreme Medical Chamber from 2021, assuming that orthopedics is a deficit specialization in Poland and that the increase in these specialists was insignificant within 4 years, statistically, for 1 orthopedist in Poland, there are 12.7 hip arthroplasty procedures and 6.2 knee arthroplasty procedures per year. Considering the ECDC data for 2017, there are 10.5 hip arthroplasty procedures and 11.2 knee arthroplasty procedures per UK orthopedist^6,12^. A comparison of these data may explain the difference in prosthetic infection rates achieved in our study compared to ECDC SSI data. Although based on the above data, we can assess a similar level of experience achieved by orthopedists in the field of hip arthroplasty, in the field of knee arthroplasty, a single surgeon will perform statistically half as many procedures in Poland as in the UK.

Total arthroplasty, compared to partial, in the obtained results was a factor that reduced the risk of SSI in the later post-surgical period. Lora-Tamayo et al. indicate the problem of early and late SSIs, where partial prostheses are characterized by a higher incidence of infection in the early period compared to full prostheses, but the author does not distinguish whether it involves superficial or deep infections^13,14^. Therefore, he emphasizes that the characteristics of the patients and their loads are also important due to the assumption of full prosthesis in stronger people, who predict increased activity in the future. Our study does not provide information on the type of injury, while many authors point to better results of total arthroplasty instead of partial, after femoral neck fracture, which could explain the obtained results especially considering that femoral neck fractures is more common in elderly^15^. Abdulkarim et al. in the review and meta-analysis indicates that there is no relationship between cemented and cementless prosthesis and the risk of infection. However, he points out that for prosthesis with antibiotic-free cement, this risk could be 1.8 times greater^16^. Konan et al., discussing the choice of the correct method of prosthesis fixation, emphasizes that cement prostheses must be used in patients with a better cardiological condition due to bone-cement implantation syndrome in cardiological unstable patients^17^. Therefore, better results for cement prostheses in HPRO may be dictated by the better general condition of the patients qualified for this method. Thus, Lentino emphasizes that in the case of revision—especially for infectious reasons—reimplantation with a cement prosthesis with an antibiotic brings better results^18^.

Older age is a typical patient-related factor associated with SSI^19,20^. The results of the cross-sectional and population study Survey of Health, Aging and Retirement in Europe (SHARE) revealed significant differences in the profile of elderly people with disabilities between European regions and Poland, Estonia, the Czech Republic, Belgium, and Portugal with higher disability quartiles in the second group despite gender or age^21^. However, we did not observe an increased risk of SSI in patients over 65 years of age, neither after HPRO nor after KPRO. This is probably because Polish patients are approximately 3–4 years younger than patients who undergo the same procedures in other countries^2^. In addition, we observed some age-gender relationship among patients with complications of SSI. The incidence of SSI in men is higher than in women, even though they were much younger than women. Age-related changes in the skin and other tissues increase the risk of impaired wound healing after surgery^22^. However, the rate of human aging is not uniform due to genetic and environmental factors. Important factors accelerating epigenetic aging are lifestyle aspects, some chronic diseases (cardiovascular diseases, cancer, diabetes, chronic obstructive pulmonary disease, depression, schizophrenia) and demographic elements (male gender, education)^23^. Such factors can modulate the rate of aging and disability in different populations. In our previous study of patient demographics, more than 60% of patients are on ATC group “M” (the musculo-skeletal system) medications, so some of these patients have e.g. rheumatic conditions that may affect the rate of SSI^2^.

In terms of diseases that predispose to increased susceptibility to infections, the results are in line with the expectations of patients with gastrointestinal diseases due to the presence of diabetic patients in this group. Research clearly shows an increased risk of infectious complications among people with diabetes. In a study by Jia-Ming Liu et al. and in the meta-analysis by Meng et al., the risk of SSI after lumbar spine surgery in patients with diabetes mellitus was more than doubled^24^. Similarly, diseases of the musculoskeletal and nervous systems affect the ability to rehabilitate patients and thus restore the body to proper functioning^25,26^. Thus, both outpatient and inpatient rehabilitation for HPRO and outpatient rehabilitation for KPRO showed a statistically significant reduction in the risk of infection in endoprostheses.

In a study by Aghdassi et al. the SSI incidence was analyzed in relation to sex, obtaining, like our study, a higher incidence in men for orthopedic surgery^27^. According to the authors of that article, it was associated with a greater number of risk factors among men, such as the percentage of staphylococcal colonization among men^28^.

It is undoubted that the center's experience is associated with a lower risk of post-surgical complications. Thresholds for the division of groups into equal numbers were defined, separating three groups. The number of operations performed directly affects the experience, which is confirmed by the authors of other publications, therefore centers performing < 100 operations were classified as the least experienced, and thus associated with the highest risk of infection^29–31^. Equally frequent infections in the most experienced centers (assessed as those performing > 200 procedures per year) may be associated with academic centers, which in the publication showed a higher risk of infection. This is due to the division of centers in Poland into three reference levels, of which academic centers are characterized by receiving the highest reference level, so it is the center most often chosen as a place of surgery. The presence of students in academic centers who, in Polish conditions, enter only for a part of the procedure generating a higher enter/exit rate may be associated with an increased risk of SSI, as evidenced by numerous guidelines evaluating the enter/exit rate as an important parameter in infection control in the operating theater^32,33^. Anderson et. al. similarly obtained more SSI for small and the largest centers, explaining this by the variability of many factors, from the experience of surgeons through cases coming to the center due to reference and treatment profile^34^. Thus, in this study, the lowest rate of SSI incidence was shown to be in centers that perform knee and hip arthroplasty in the amount of 100–200 for each of these procedures.

In the obtained results, patients hospitalized in the intensive care unit (ICU) had up to ten times higher risk of deep SSI. According to the publication by Al-Mulhim et al., based on a 5-year analysis of orthopedic patients in the field of SSI, this is due to the difference in the pathogens characteristic for the ICUs and other. In their research, they showed that Acinetobacter spp. was responsible for SSI as the second most common pathogen and therefore is a strain commonly found in ICUs^35^. In our study, we did not receive detailed data on the reason for hospitalization in the ICU; however, the median TISS score for these patients for both procedures were 40.0, so, it can be assumed that the patient's condition was serious and required significant interventions (including intubation, more intravenous accesses, central lines etc.) to maintain vital functions in conditions of the ICU.

Antibiotics administered at discharge in studied population increased the risk of detection of SSI after HPRO and KPRO by up to 4 and 7 times, respectively. This may be a proof of detection of the first lightly symptoms of SSI already at discharge, most probable explanation is that it may represent cases of superficial SSIs or other infection with mild symptoms, diagnosed by physicians. This may indicate lack of effective infection control in Polish hospitals, including lack of reliable detection and registration that results in a much smaller than expected number of superficial SSIs, reported in the earlier polish studies^10,11^.

The response of orthopedic surgeons to the growing problem of prosthesis infections was the first and second Philadelphia consensus, in which the most important elements affecting the risk of infection were collected. Nevertheless, as the authors of the Polish study emphasize, experts in many fields had divergent opinions, which implied the need for further research^36^.

Due to retrospective character of the study based on anonymized database, there were lot of limiting factors, including lack of information about duration of the surgery, occurred complications at intraoperative period and surgical antibiotic prophylactic—one of the most important element in infection prevention. In Poland, according to experts' recommendations, perioperative prophylaxis is administered—1 g of cefazolin in patients < 80 kg or 2 g of cefazolin in patients > 80 kg—however, the provided database does not contain information on its use in everyday clinical practice. Also due to anonymization and lack of sample connection with patients, there was no possibility to analyze the microbial etiology of the infection.

## Conclusions

The incidence of SSI in the Polish population is probably much higher than in other European countries that perform a similar number of hip and knee arthroplasty procedures, but there is a need to prospective analysis of both in-hospital and post-discharge SSI to confirm this observation. The study shows that special attention should be paid to patients with various chronic diseases—as factors increasing risk of SSI—especially those affecting the possibility of applying a protective rehabilitation, because lack of rehabilitation in pre-surgery and post-surgery period was one of the strongest deteriorating factor. The risk was extremally modified to the disadvantage for ICU hospitalized and discharged with antibiotics—these patients should receive special care during the post-discharge period.

Due to their impact on the health and life of patients, the presented results can be an important voice in the discussion of prevention and control of SSI and, above all, confirm the urgent need of common implementation of an effective, based on recommendations, infection control program including surveillance of SSI that covers patients undergoing arthroplasty in Polish hospitals.

### Supplementary Information

Supplementary Information 1.

Supplementary Information 2.

## Data Availability

The data underlying this article will be shared on reasonable request to the corresponding author.

## Abbreviations

ATC: Anatomical Therapeutic Chemical Classification
ECDC: European Center of Disease Prevention and Control
HAI: Hospital acquired infection
HPRO: Hip replacement surgery
ICU: Intensive care unit
KPRO: Knee replacement surgery
NFZ: Narodowy Fundusz Zdrowia/National Health Fund
OECD: Organisation for Economic Cooperation and Development
SSI: Surgical site infection
UK: United Kingdom
