# Supplementary material for: Post-discharge occurrence of surgical site infections after hip or knee arthroplasty surgery in Poland, a population-based study
Source: Sci Rep. 2023 Sep 24;13:15940. doi: 10.1038/s41598-023-43111-z (PMC10518305; doi:10.1038/s41598-023-43111-z)
Supplement: Supplementary file 1 — Supplementary Information 1. [file 41598_2023_43111_MOESM1_ESM.docx]

APPENDIX II

The following appendix includes statistical data used in the article in the form of text to avoid duplication of data in the form of tables and text.

Table 1S Statistical analysis of hospital data

|  | | HPRO | KPRO |  |
| --- | --- | --- | --- | --- |
| Outpatients Clinic days to diagnose | | 41.0 (21.0; 59.0) | 44.5 (22.0; 74.0) | 0.097 UMW |
| Hospital days to diagnose | | 24.0 (13.0; 51.0) | 33.0 (25.0; 74.0) | 0.179 UMW |
| Outpatients clinics | Primary center | 198 (63.3%) | 99 (62.7%) | 0.979 Chi2 |
|  | Other | 115 (36.7%) | 59 (37.3%) |  |
| Hospital | Primary center | 20 (74.1%) | 5 (62.5%) | 0.661 Fisher |
|  | Other | 7 (25.9%) | 3 (37.5%) |  |
| Discharge-readmission time [days] | | 33.00 (19.5; 56.5) | 42.0 (28.0; 62.0) | 0.002 UMW |
| Site | Same as primary | 247 (93.56%) | 106 (93.81%) | 1.000 Chi2 |
|  | Other | 17 (6.44%) | 7 (6.19%) |  |
