# Supplementary material for: Post-discharge occurrence of surgical site infections after hip or knee arthroplasty surgery in Poland, a population-based study
Source: Sci Rep. 2023 Sep 24;13:15940. doi: 10.1038/s41598-023-43111-z (PMC10518305; doi:10.1038/s41598-023-43111-z)
Supplement: Supplementary file 2 — Supplementary Information 2. [file 41598_2023_43111_MOESM2_ESM.docx]

**Appendix**

**Data provided in the form of a collection from the National Health Fund:**

1. Patient data:

a) Gender

b) Age

c) Place of residence: village, town below 100,000 residents, a city with over 100,000 residents

d) Was the patient vaccinated (up to definition) in the year before the operation due to:

- - 1. HBV; vaccination at any time (depending on the archival data of the National Health Fund), at least 14 days before the procedure
    2. flu:
       1. surgery performed during the flu season, i.e. on October 1-March 31: vaccination should take place on September 1 of the preceding year - up to 7 days before surgery
       2. if the operation took place in the off-season: i.e. April 1 - September 30 - there were no indications for vaccination

e) Rehabilitation within 90 days prior to surgery, as defined in ICD-9

f) Whether the patient suffered from selected chronic diseases, defined on the basis of purchased drugs, acc. ACT code, respectively in groups:

- - 1. drugs used in diseases of the digestive system, including diabetes: A02BC, A10 A10B, A10B
    2. drugs acting on the hematopoietic system: B01: B01AA / B01AB / B01AC / B01AD / B01AE / B01AF / B01AX, B03
    3. drugs in cardiovascular diseases: C01 / C02 / C03 / C04 / C05 / C06 / C07 / C08 / C09 / C010,
    4. hormonal drugs: G03, H01 / H02 / H03 / H04 / H05
    5. anti-cancer drugs: L01 / L02 / L03 / L04
    6. drugs acting on the musculoskeletal system: M01 / M02 / M03 / M04 / M05 / M09
    7. drugs acting on the nervous system: N01 / N02 / N03 / N04 N04 / N06 / N07
    8. drugs affecting the respiratory system: R01 / R02 / R03 / R04 / R05 / R06 / R07
    9. polytherapy: taking 5 drugs or more regardless of the number of groups from how many of the above groups does the patient take drugs: i.e. 0 or 2, 3 etc.

2. Entity carrying out the procedure:

a) Is it a learning unit

b) Number of arthroplasty: knees / hips that the unit performed in the year before surgery

c) Distance from the patient's place of residence

1. Information about arthroplasty:

a) primary / revision / revision without replacing elements

b) partial / total

c) cemented / cementless

1. Data on hospitalization related to arthroplasty:

a) Mode of admission (emergency or scheduled)

b) Reason for hospitalization according to ICD-10 codes

c) Time from admission to hospital to surgery

d) Was the procedures defined in accordance with ICD-9 during the operation

e) Time from surgery to discharge from the department where the operation was performed

f) What was the ward to which the patient was transferred from the ward where the operation was performed (according to Part VIII of the departmental code)

- - 1. Length of stay in this ward
    2. If it was an anaesthesiology and intensive care unit, the maximum TISS value from this stay

g) Time from discharge from the ward to discharge from the hospital

h) Was hospitalization fatal?

1. Have drugs from the following groups been prescribed on discharge (date of order equal to the date of discharge)

a) defined as redemption, according to ATC code:

- - 1. appropriate drug, according to ATC code: M01 / M02 / M03 / M04 / M05 / M06 / M07 / M08 / M09, H05
    2. all antibiotics, i.e. all J01 drugs

1. Infections after surgery

a) Was the patient treated within 90 days of surgery for infection and inflammation caused by an internal joint prosthesis (ICD-10: T84.5)

b) Was the patient provided services for lower respiratory tract, urinary tract, digestive and other infections defined by the ICD-10 within 30 days of the operation?

1. For all patients, data on post-operative hospitalization services

a) Have any inpatient rehabilitation services been provided within 42 days of the operation?

b) Have any outpatient rehabilitation services been provided within 42 days of the operation?

c) Did the patient receive benefits at care and treatment facility (National Health Fund(NFZ): based on concluded contracts) within 90 days from the operation

d) Has the patient used a long-term care nurse (NFZ: on the basis of concluded contracts) within 90 days from the operation

1. For all patients, if the patient died during hospitalization within 120 days of surgery

1. For patients who received services for the reasons listed in point 6, data on services after hospitalization related to the operation (up to 120 days from the date of surgery)

a) Admission to the ward

- - 1. Reason for admission according to ICD-10
    2. Distance from the patient's place of residence
    3. Department to which the patient was admitted: conservative / interventional / anesthesiology and intensive care unit (if it was an anesthesiology and intensive care unit, the maximum TISS value from this stay)
    4. Was the prosthesis revision performed (definition according to ICD-9 codes)
    5. Is it the same center that performed the arthroplasty?
    6. length of hospitalization
    7. whether secondary hospitalization resulted in the patient's death
    8. Hospitalization cost

b) Admissions to the Emergency Department

- - 1. Reason
    2. Is it the same site that performed the operation?

c) Outpatient treatment or primary health care

- - 1. Reason f
    2. Is it the same site that performed the operation?
    3. Was it an internal medicine practitioner / geriatrician/ orthopaedist / general practitioner, defined on the basis of part VIII of the departmental code

d) Prescriptions after surgery (order date greater than the date of discharge, up to 30 days from the date of surgery): each prescription with a direct reference to the procedure performed, i.e. demographic details, details of the prescription

- - 1. whether there was a drug from a given group, only antibiotics: all drugs from the J01 group with the specification of the substance (according to codes e.g. J01CR02)
    2. the amount of the substance (or information on the number of packages with the dose in the package)
    3. date of issue of the prescription
    4. demographic stratification of patients taking into account groups of patients classified according to the operating procedure.

**Abbreviations:**

ATC- Anatomical-therapeutic-chemical classification, HBV- Hepatitis B Virus, NFZ-National Health Found, TISS-Therapeutic intervention scoring system,
